# In reply to “Comment on “Particle therapy using protons or carbon ions for cancer patients with cardiac implantable electronic devices (CIED): a retrospective multi-institutional study””

**DOI:** 10.1007/s11604-022-01253-6

**Published:** 2022-02-19

**Authors:** Takayuki Hashimoto

**Affiliations:** Department of Radiation Medical Science and Engineering, Faculty of Medicine, Hokkaido University, Street address: North 15 West 7, Kita-ku, Sapporo, Hokkaido 060-8638 Japan

To the Editor,

We are grateful for the detailed and valuable comments in response to our article [1]. We cited the report of Matsubara et al. in the original paper, where they describe the probability of developing cardiac implantable electronic device (CIED) errors [2]. In the Matsubara Letter to the Editor [3], they stated that: (a) if the same physical dose is irradiated, carbon ion therapy (CIT) is safer than proton beam therapy (PBT) even without considering relative biological effectiveness (RBE), because the number of particles itself and the generated number of secondary neutrons are much smaller in CIT, (b) the reaction cross section of the neutron beam production is larger for carbon beams, CIT is still safer than PBT, however. We agree with their observation as we described: “Also, the previous dosimetric studies suggested that CIT generates fewer secondary neutrons than PBT even for the same physical absorbed dose in Gy” referring to the Matsubara et al. paper in the Discussion section of the original paper [1]. However, probably due to our insufficient explanation, our phrasing seems to have been too vague to be understood correctly. We did not mean that RBE is the only reason for the fewer secondary neutrons. We agree that RBE could be a minor reason comparing to the fewer secondary neutrons in CIT than in PBT as explained beautifully by Matsubara [3].

Using this opportunity, we would like to address and stress the following issues again. The generation of neutrons that affect CIED is not determined only by the type of radiation, the number of accelerated particles, the reaction cross section, or the RBE. At present, we should consider many other factors such as scanning or passive irradiation, the direction of beam injection, the irradiation field formation system, and internal scattering. In practice, since neutrons are not easily measured, it is important to carefully and logically identify and isolate the factors that cause CIED errors to be able to reduce these. The main focus of our study was not the verification of the logical process, the suggestions by Matsubara have shed light on the importance of the logic again. We believe that continuous and continuing collaboration between physicians and physics researchers in particle beam facilities is critically important to find and transmit evidence to the world.
